# Metabolomic Profile of Posner–Schlossman Syndrome: A Gas Chromatography Time-of-Flight Mass Spectrometry-Based Approach Using Aqueous Humor

**DOI:** 10.3389/fphar.2019.01322

**Published:** 2019-11-07

**Authors:** Haiyan Wang, Ruyi Zhai, Qian Sun, Ying Wu, Zhujian Wang, Junwei Fang, Xiangmei Kong

**Affiliations:** ^1^Department of Ophthalmology, Shanghai General Hospital, Shanghai Jiao Tong University School of Medicine, Shanghai, China; ^2^Shanghai Key Laboratory of Ocular Fundus Diseases, Shanghai Jiao Tong University, Shanghai, China; ^3^Shanghai Engineering Center for Visual Science and Photomedicine, Shanghai, China; ^4^Department of Ophthalmology and Visual Science, Eye, Ear, Nose and Throat Hospital, Shanghai Medical College, Fudan University, Shanghai, China; ^5^Key Laboratory of Myopia, Ministry of Health, Fudan University, Shanghai, China; ^6^Shanghai Key Laboratory of Visual Impairment and Restoration, Fudan University, Shanghai, China; ^7^College of Basic Medical Sciences, Shanghai Jiao Tong University School of Medicine, Shanghai, China

**Keywords:** Posner–Schlossman syndrome, metabolism, mass spectrometry, gas chromatography time-of-flight mass spectrometry, aqueous humor

## Abstract

The Posner–Schlossman syndrome (PSS) is a disease with clinically recurrent unilateral anterior uveitis with markedly elevated intraocular pressure (IOP) and subsequent progression to optic neuropathy. Retrospective studies have reported increased annual incidence of PSS, especially in China. While currently, the clinical management of PSS is still challenging. Metabolomics is considered to be a sensitive approach for the development of novel targeted therapeutics because of its direct elucidation of pathophysiological mechanisms. Therefore, we adopted gas chromatography time-of-flight mass spectrometry (GC-TOF-MS) technology-based non-targeted metabolomics approach to measure comprehensive metabolic profiles of aqueous humor (AH) samples obtained from patients with PSS, with an aim to demonstrate the underlying pathophysiology, identify potential biomarkers specific to PSS, and develop effective treatment strategies. A comparative analysis was used to indicate the distinct metabolites of PSS. Pathway analysis was conducted using MetaboAnalyst 4.0 to explore the metabolic reprogramming pathways involved in PSS. Logistic regression and receiver-operating characteristic (ROC) analyses were employed to evaluate the diagnostic capability of selected metabolites. Comparative analysis revealed a clear separation between PSS and control groups. Fourteen novel differentiating metabolites from AH samples obtained from patients with PSS were highlighted. Pathway analysis identified 11 carbohydrate, amino acid metabolism and energy metabolism pathways as the major disturbed pathways associated with PSS. The abnormal lysine degradation metabolism, valine–leucine–isoleucine biosynthesis, and citrate circle were considered to weigh the most in the development of PSS. The ROC analysis implied that the combination of glycine and homogentisic acid could serve as potential biomarkers for the discrimination of control and PSS groups. In conclusion, these results revealed for the first time the identity of important metabolites and pathways contributing to the development/progression of PSS, enabled the better understanding of the mechanism of PSS, and might lead to the development of metabolic biomarkers and novel therapeutic strategies to restrict the development/progression of PSS.

## Introduction

Posner–Schlossman syndrome (PSS), also called glaucomatocyclitic crisis (GCC), is a disease with clinically recurrent unilateral anterior uveitis with markedly elevated intraocular pressure (IOP) and subsequent progression to optic neuropathy (Eissler, 1948; Moorthy et al., 1997). Retrospective studies have reported increased annual incidence of PSS, especially in China (Jiang et al., 2017). The clinical management of PSS is still challenging. Interventional measure for PSS is indicated for managing the associated inflammation and elevated IOP in order to prevent optic neuropathy. The use of corticosteroid can relieve the early stage symptoms; however, it fails to prevent the recurrence of PSS. Moreover, many patients develop corticoid dependence over shorter intervals gradually, complicating the management of PSS. Long-term administration of ganciclovir may control the symptoms of certain types of PSS (Miyanaga et al., 2010; Sobolewska et al., 2014; Su et al., 2014). However, the patients still experience progressive endothelium loss and a high relapse rate after the withdrawal of the drug (Moorthy et al., 1997; Chee and Jap, 2010; Miyanaga et al., 2010; Sobolewska et al., 2014; Su et al., 2014; Jiang et al., 2017). Eventually, repeated uncontrolled elevation of IOP can result in irreversible optic neuropathy and vision loss. Better understanding of the etiology and pathophysiological mechanism of PSS may aid in blunting the pathogenesis/progression of this disease. Several risk factors for PSS have been reported, such as inflammatory cytokines (Ohira et al., 2016; Pohlmann et al., 2018), vascular endothelial dysfunction (Shen et al., 2010), and primarily, human cytomegalovirus (HCMV) infection (Teoh et al., 2005; Chee and Jap, 2008; Van Gelder, 2008; Shazly et al., 2011; Su et al., 2014), which tops the list. Studies have demonstrated the participation of various metabolites in the inflammation process (Tannahill et al., 2013; Mills and O'Neill, 2014), including the HCMV-induced inflammatory response. Metabolites, as the downstream products of gene transcription, translation, and post-translational protein modification, are directly influenced by the physiological and pathological changes in tissues and accurately reflect the physiological changes (Oldiges et al., 2007; Baharum and Azizan, 2018). Therefore, studies investigating the change in endogenous metabolites and related metabolic pathways, underlying PSS, may provide a more sensitive approach to identify the pathophysiological mechanisms and, hopefully, lead to the identification of metabolic biomarkers for the prognosis of PSS and provide novel hypotheses for developing targeted therapeutics (Baharum and Azizan, 2018; Mayerle et al., 2018).

In metabolomic studies, mass spectrometry (MS) is the most preferred technique. Gas chromatography time-of-flight mass spectrometry (GC-TOF-MS) is perfectly suited for the identification and quantitation of low molecular weight metabolites (Phua et al., 2013; Jaeger et al., 2017; Yin et al., 2017) because of its key features, including higher sensitivity, selectivity, resolution, and accuracy of detection. Therefore, we adopted GC-TOF-MS technology-based non-targeted metabolomics approach and multivariate statistical analysis to measure comprehensive metabolic profiles of aqueous humor (AH) samples obtained from patients with PSS and cataract, with an aim to demonstrate the underlying pathophysiology, identify potential biomarkers specific to PSS, and develop effective treatment strategies.

## Methods

### Research Design

The study was prospectively approved by the medical ethics committee of the Eye, Ear, Nose and Throat (EENT) Hospital of Fudan University (2017006–2), and research was conducted in accordance with the Declaration of Helsinki as revised in 2000. Signed informed consent was obtained from all participants enrolled in the study. We conducted this prospective, observational, and internal research project from June 2018 to December 2018 at EENT Hospital of Fudan University.

PSS was diagnosed based on the following clinical features: a) unilateral recurrent mild iridocyclitis; b) nonpigmented keratic precipitates (KPs) and corneal edema; c) cell and flare in the anterior chamber; d) elevated IOP; e) no posterior synechiae or peripheral anterior synechiae and posterior inflammation; and f) a relatively short attack duration. All participant data including gender, age, IOP, best-corrected visual acuity (BCVA), cornea endothelial cell density (CD), KP, Tyndall, and vertical ratio of cup to disk (C/D) were recorded and documented in the electronic case report form. In total, 43 participants were enrolled for this study, including 12 participants with PSS and 12 participants with cataract. All the PSS patients enrolled in this study were naïve patient without any treatment. All the PSS patients were at the acute onset stage.

### Sample Collection

Paracentesis of anterior chamber was performed to collect AH sample for MS and anti-virus IgG concentration analysis. Meanwhile, serum sample was obtained in our central laboratory with the use of standardized procedures.

### Analysis of Anti-Virus IgG Concentration in AH

Anti-virus IgG in AH and serum were detected by enzyme-linked immunosorbent assay (ELISA) kit (Virion\Serion, Germany). The test procedure was performed according to the kit instructions. Albumin was detected by scattering immunonephelometry (Guosai Biotechnology Co., Ltd, Shenzhen, China). The concentration of anti-virus IgG in AH was presented by a corrected ratio of (AH IgG/serum IgG) to (AH albumin/serum albumin), which was abbreviated as s/co. Mann–Whitney *U* test was performed to measure the significance of anti-virus IgG concentration between PSS and control groups.

### Correlation Study Between Anti-Virus IgG Concentration and Clinical Variables

Spearman’s rank correlation coefficient was performed to evaluate whether a correlation existed between both variables (anti-virus IgG concentration and clinical variables). Spearman’s rank correlation coefficient value (Spearman’s rho) ranged from 0 (no reliability) to 1 (perfect reliability). *p* values ≤ 0.05 were considered significant. All the analyses were carried out using SPSS 22.0 statistical software.

### Mass Spectrometry Analysis

AH sample aliquots of 0.15 ml were transferred into cryovial tubes and stored at −80°C immediately. An Agilent 6890N gas chromatograph (Agilent Technologies) coupled to a Pegasus HT TOF MS (LECO Corp., St. Joseph, MI, USA) was used as the GC-TOF-MS platform. The sample preparation procedure and instrumental analysis were referred in the previously published methods (Qiu et al., 2014) with minor modifications, which was summarized in the **Supplementary Materials**.

### Statistical and Data Analysis

For GC/MS data, data processing and identification are in accordance with our previously published work using XploreMET (Pan et al., 2010). The resulting data were exported into Microsoft Excel, and the peaks were normalized to the total sum of spectrum prior to multivariate analyses. The normalized data were analyzed by SIMCA-P (version 14.0 Umetrics AB, Umea, Sweden) for unsupervised principal component analysis (PCA) to obtain a general overview of the variance of metabolic phenotypes among different groups. In addition, supervised orthogonal projection to latent structure-discriminant analysis (OPLS-DA) was performed to obtain information about the variance of metabolic phenotypes that correspond to the classes. Student’s *t*-test with Bonferroni’s correction and fold change were performed afterwards to evaluate the significance of each metabolite. Significantly changed metabolites were identified by the parameters of OPLS-DA VIP > 1, *t*-test *p* < 0.05, and fold change > 1.2 or <0.83. The correlation between significantly disturbed metabolites and clinical variables of PSS patients was investigated by Spearman’s rank correlation analysis using MetaboAnalyst 4.0 (http://www.metaboanalyst.ca/) (Xia and Wishart, 2016).

Pathway analysis was conducted using MetaboAnalyst 4.0 as well as the Kyoto Encyclopedia of Genes and Genomes (KEGG) database (www.genome.jp/kegg/). Former collected metabolites were used as input. A logistic regression analysis and receiver-operating characteristic (ROC) analysis were performed using SPSS software version 18.0 (IBM Corp., Armonk, New York) to evaluate the diagnostic capability of selected metabolites. The area under the ROC curve (AUC) was calculated to quantify the performance of the diagnostic variables.

## Results

### Sample Characteristics

The clinical characteristics of participants selected for discovery metabolomic profiling are shown in **Table 1**.

**Table 1:** Clinical characteristics of participants in PSS and control groups.

	PSS	Control	*p* value
Number	12	12	
Gender (male, %)	5, 41.7	4, 33.3	0.673Ψ
Age (years)	51.3 ± 9.9	58.3 ± 7.9	0.072
IOP (mmHg)	21.6 ± 9.4	14.3 ± 2.6	0.02*
BCVA	3.64 ± 1.9	3.93 ± 0.7	0.687
CD	2704.6 ± 190.9	2626.7 ± 282.3	0.486
KP (y/n)	12/0	0/12	<0.001Ψ**
Tyndall (y/n)	3/9	0/12	0.064Ψ
Vertical C/D	0.4 (0.3–0.9)	0.3	<0.001#***

PSS, Posner–Schlossman syndrome; BCVA, best-corrected visual acuity; IOP, intraocular pressure; CD, cell density; KP, keratic precipitate; C/D, ratio of cup to disk. # by Mann–Whitney U test; Ψ, by χ^2^ test. *, p value < 0.05; **, p value < 0.01.

### Anti-Virus IgG Concentration in AH

There was a significant difference of anti-HCMV IgG concentration between PSS and control groups (*p* value < 0.001) **Table 2**.

**Table 2:** Anti-virus IgG concentration in AH from PSS and control groups.

	PSS	Control	*p* value
Number	12	12	
Anti-HCMV IgG (s/co)	0.45 (0.12–2.39)	0.01 (0–0.02)	<0.001***
Anti-rubella virus IgG (s/co)	0.03 (0.01–0.08)	0.01 (0.01–0.03)	<0.001***
Anti-VZV IgG (s/co)	0.02 (0.01–5.08)	0.01 (0–0.01)	0.024*
Anti-HSV1 IgG (s/co)	0.01 (0.01–0.04)	0.01 (0.01–0.02)	0.713

PSS, Posner–Schlossman syndrome; HCMV, human cytomegalovirus; VZV, varicella zoster virus; HSV, herpes simplex virus. Data were presented as median (range). Mann–Whitney U test was used to compare the anti-virus IgG concentration between PSS group and control group. *, p value < 0.05; ***, p value < 0.001.

### Correlation Analysis Between Anti-Virus Igg Concentration and Clinical Manifestations

In **Table 3**, Spearman’s rank correlation coefficient indicated high agreement of HCMV and IOP (*r* = 0.458, *p* < 0.05), HCMV and C/D (*r* = 0.828, *p* < 0.001), and HCMV and KP (*r* = 0.879, *p* < 0.01). Statistical correlation was also found between VZV and Tydall (*r* = 0.586, *p* < 0.01), VZV and C/D (*r* = 0.426, *p* < 0.05), and VZV and KP (*r* = 0.533, *p* < 0.01).

**Table 3:** Spearman's correlation between anti-virus IgG concentration and clinical indexes.

	IOP	CD	CCT	C/D	KP	Tydall
Anti-HCMV IgG (s/co)	0.458*	−0.067	0.600	0.828***	0.879***	0.296
Anti-VZV IgG (s/co)	0.202	−0.174	−0.516	0.426*	0.533**	0.586**
Anti-rubella virus IgG (s/co)	0.233	−0.228	−0.261	0.535*	0.689***	0.563**
Anti-HSV1 IgG (s/co)	0.121	−0.501*	0.131	0.091	0.119	0.167

Correlation coefficient and p values are calculated by Spearman’s correlation. HCMV, human cytomegalovirus; VZV, varicella zoster virus; HSV, herpes simplex virus; IOP, intraocular pressure; CD, cell density; CCT, central corneal thickness; C/D, ratio of cup to disk; KP, keratic precipitate. *, p value < 0.05; **, p value < 0.01; ***, p value < 0.001.

### Global Metabolomic Profiles Between PSS and Control Groups

**Figure 1:**
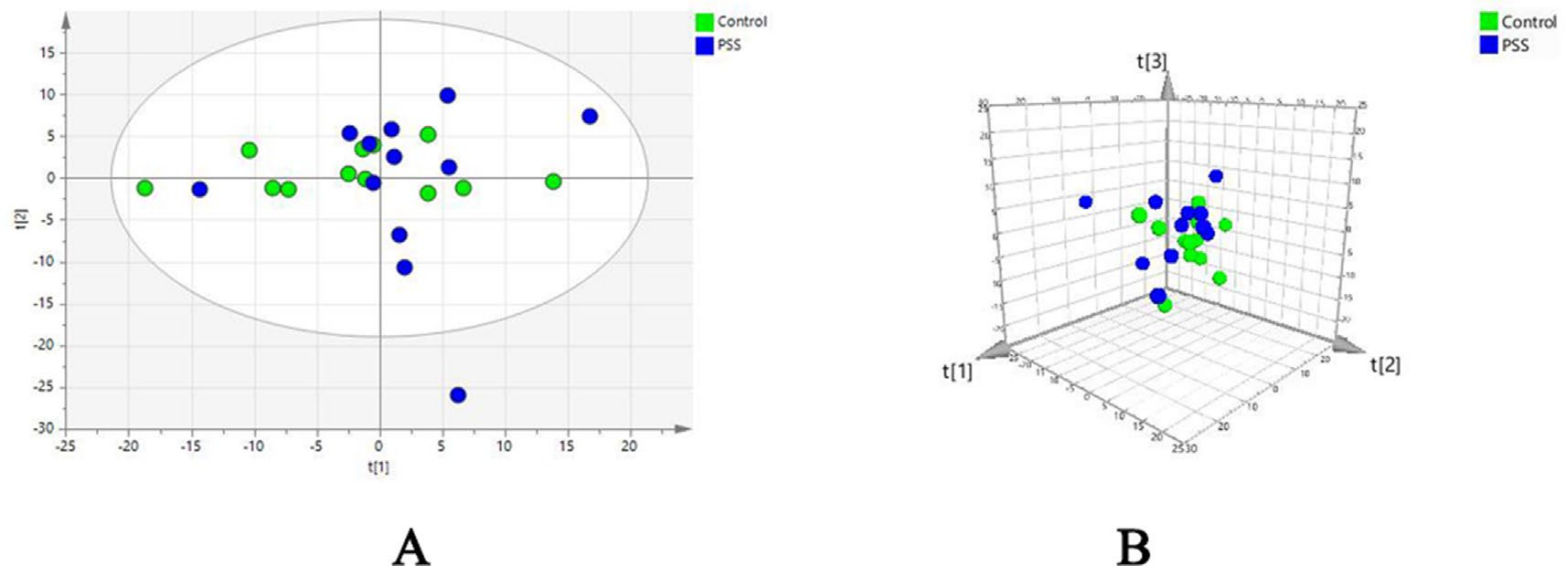
Principal component analysis. Principal component analysis scatter plots showing the first two **(A)** and three **(B)** principal components with control and PSS patients. PSS, Posner–Schlossman syndrome.

Evaluation of metabolomic profiles between PSS and control groups was conducted using unsupervised statistics, PCA. The PCA score plot with the first two (**Figure 1A**) and three (**Figure 1B**) principal components demonstrated a clear separation between the PSS and control groups, indicating that PSS caused gradual alterations in metabolism.

### Significantly Disturbed Metabolites Between PSS and Control Groups

**Figure 2:**
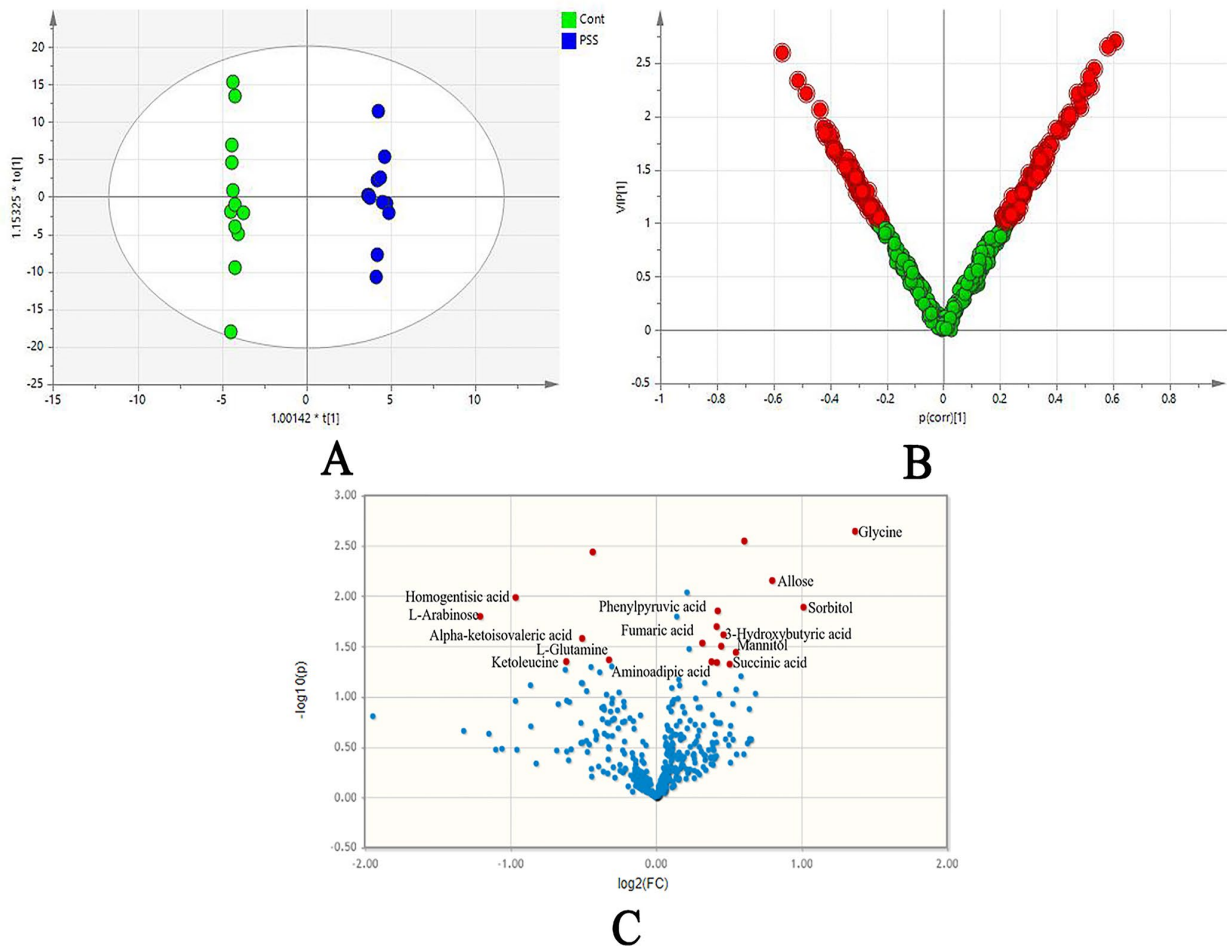
The orthogonal projection to latent structure-discriminant analysis (OPLS-DA). OPLS-DA score plots **(A)** showed clear differences obtained for PSS versus control groups; loading plot **(B)** presented significantly disturbed metabolites in red (VIP 1); volcano plot **(C)** illustrated metabolites with their association with PSS, demonstrated as fold difference (*x*-axis) and *p* value (*y*-axis). PSS, Posner–Schlossman syndrome.

In the OPLS-DA model, clear differences were obtained for PSS versus control groups: cumulative R2Y at 0.995 and Q2Y at 0.445 (**Figure 2A**). VIP values and correlation coefficients (i.e., p[corr]) of each metabolite was shown in the V-plot, and 136 variables in red were found to have VIP values > 1 (**Figure 2B**). Metabolites with significant changes were easily isolated with the help of volcano plot, shown in **Figure 2C**. The permutation test results of OPLS-DA model are shown in **Figure S1**. The plot demonstrated as fold difference (*x*-axis) and *p* value (*y*-axis). Fourteen metabolites with significant abundance changes were defined and selected by the parameters of OPLS-DA VIP > 1, *t*-test *p* < 0.05, and fold change > 1.2 or <0.83 (**Table 4**).

**Table 4:** Significantly disturbed metabolites.

Name	VIP	*p* value	Fold change
3-Hydroxybutyric acid	1.739	0.024	1.38
Allose	1.809	0.007	1.74
Alpha-ketoisovaleric acid	1.531	0.026	0.704
Aminoadipic acid	1.539	0.045	1.303
Fumaric acid	1.68	0.02	1.336
Glycine	2.104	0.002	2.582
Homogentisic acid	1.916	0.01	0.513
Ketoleucine	1.519	0.045	0.653
L-Arabinose	1.659	0.016	0.433
L-Glutamine	1.649	0.043	0.8
Mannitol	1.632	0.036	1.464
Phenylpyruvic acid	1.72	0.014	1.343
Sorbitol	1.865	0.013	2.02
Succinic acid	1.425	0.047	1.421

### Spearman’s Rank Correlation Analysis Between Disturbed Metabolites and Anti-Virus IgG Concentration in AH

**Figure 3:**
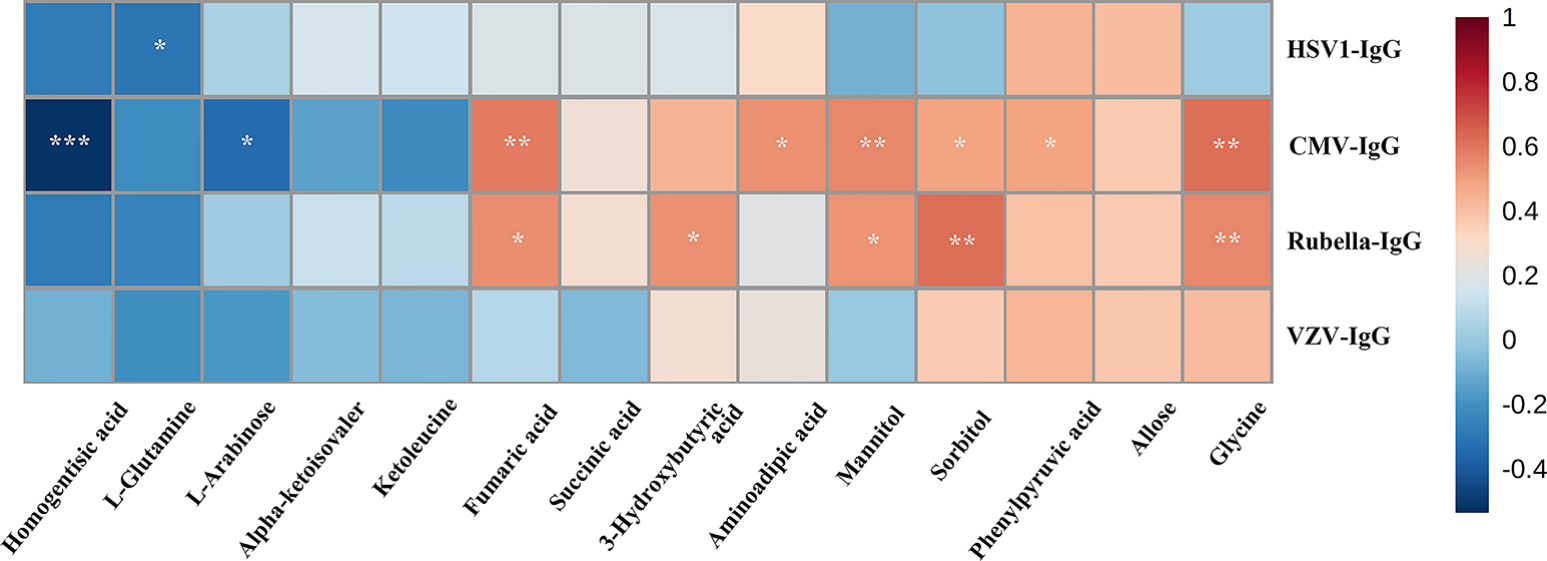
Spearman’s rank correlation analysis. Spearman’s rank correlation analysis performed between 14 differential metabolites and anti-virus IgG concentration. Red, positive correlation; blue, negative correlation. CMV, cytomegalovirus; VZV, varicella zoster virus; HSV, herpes simplex virus. *, adjusted *p* value 0.05; **, adjusted *p* value 0.01, ***, adjusted *p* value 0.001.

Spearman’s rank correlation analysis indicated that there was strong positive correlation between anti-HCMV-IgG and glycine, phenylpyruvic acid, sorbitol, mannitol, aminoadipic acid, fumaric acid (FA), and 3-hydroxybutyric acid. Meanwhile, homogentisic acid and -arabinose exhibited notably negative correlation with anti-HCMV-IgG concentration (**Figure 3**).

### Metabolic Pathway Analysis Associated With PSS

**Figure 4:**
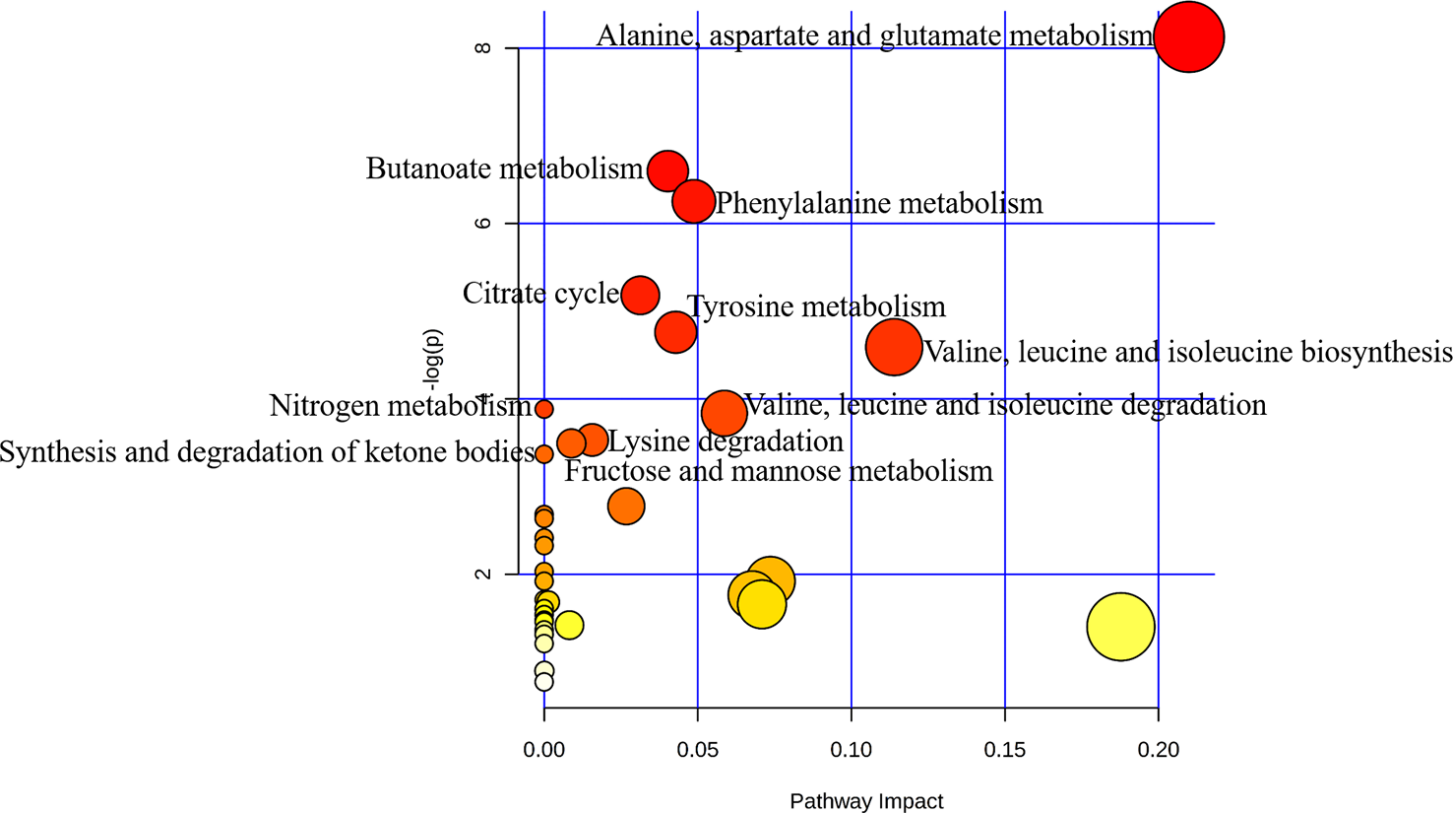
Pathway analysis of metabolites with significant difference using MetaboAnalyst. Bubble area is proportional to the impact of each pathway, with color denoting the significance from highest in red to lowest in white.

Pathways relevant to 14 differential metabolites in AH are shown in **Figure 4** and **Table 5**. The abnormal of citrate cycle, valine–leucine–isoleucine biosynthesis, valine–leucine–isoleucine degradation, butanoate metabolism, fructose and mannose metabolism, lysine degradation metabolism, alanine, aspartate and glutamate metabolism, nitrogen metabolism, phenylalanine metabolism, tyrosine metabolism, and synthesis and degradation of ketone bodies metabolism were highlighted as the most important pathways in PSS group (*p* < 0.05).

**Table 5:** Pathways relevant to 14 differential metabolites.

Pathway	Metabolites	Total	Impact	Hits	*p* value
Alanine, aspartate, and glutamate metabolism	L-Glutamine, fumaric acid, succinic acid	24	0.1396	3	0.0003
Butanoate metabolism	(*R*)-3-Hydroxybutyric acid, succinic acid, fumaric acid	40	0.2327	3	0.0014
Citrate cycle (TCA cycle)	Succinic acid, fumaric acid	20	0.1163	2	0.0056
Fructose and mannose metabolism	Sorbitol, mannitol	48	0.2792	2	0.0304
Lysine degradation	Aminoadipic acid, glycine	47	0.2734	2	0.0293
Nitrogen metabolism	L-Glutamine	39	0.2268	2	0.0206
Phenylalanine metabolism	Phenylpyruvic acid, succinic acid, fumaric acid	45	0.2617	3	0.0019
Synthesis and degradation of ketone bodies	(*R*)-3-Hydroxybutyric acid	6	0.0349	1	0.0344
Tyrosine metabolism	Homogentisic acid, fumaric acid, succinic acid	76	0.442	3	0.0086
Valine, leucine, and isoleucine biosynthesis	Alpha-ketoisovaleric acid, 4-Methyl-2-oxopentanoate	27	0.157	2	0.0102
Valine, leucine, and isoleucine degradation	Alpha-ketoisovaleric acid, 4-Methyl-2-oxopentanoate	40	0.2327	2	0.0216

TCA, tricarboxylic acid.

### Potential Biomarkers Analysis for Discrimination

**Figure 5:**
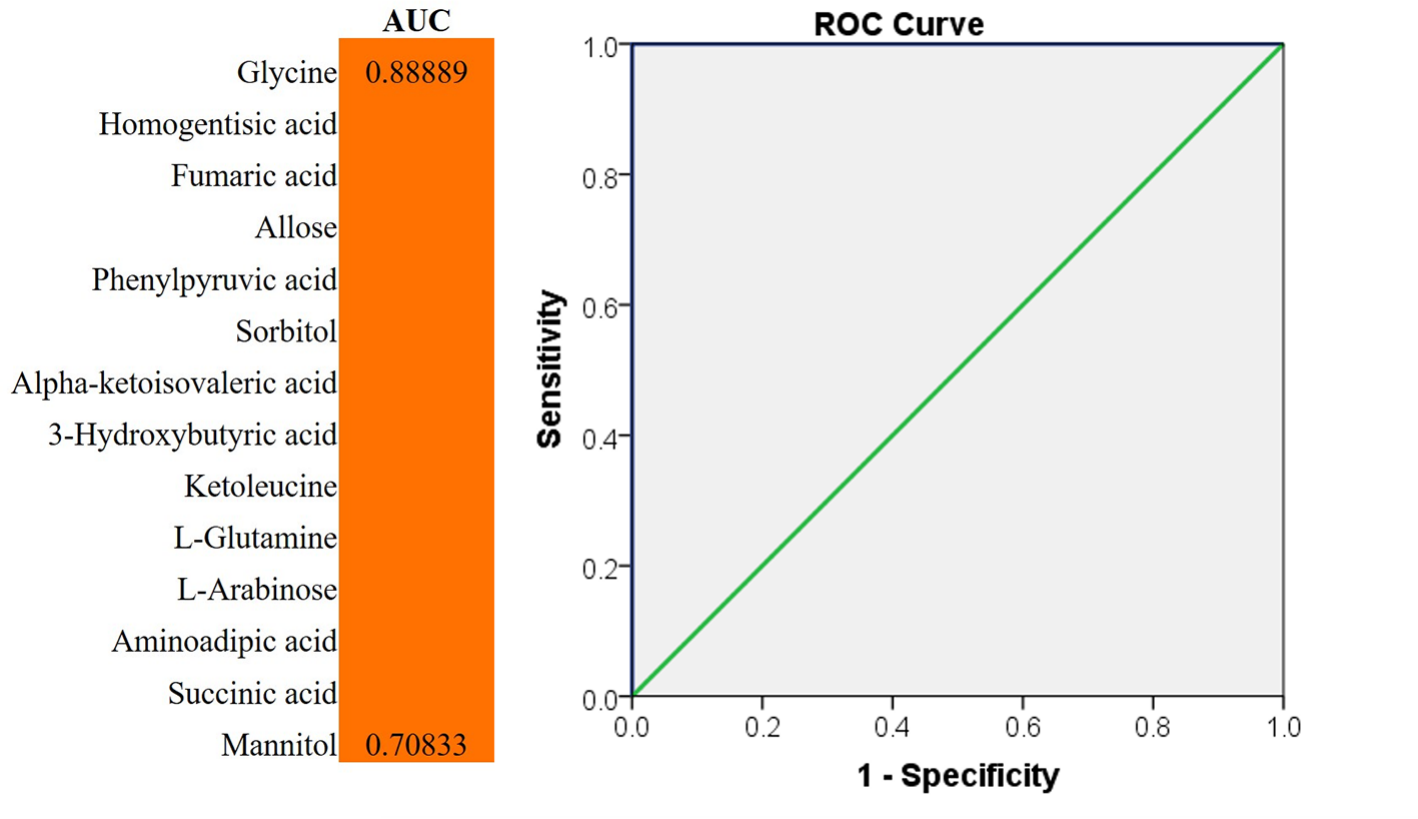
ROC analysis for discrimination. The diagnostic outcomes of potential biomarkers were shown *via* the ROC curves for comparison between PSS versus control; ROC, receiver-operating characteristic; AUC, area under the receiver operating characteristic curve.

**Figure 6:**
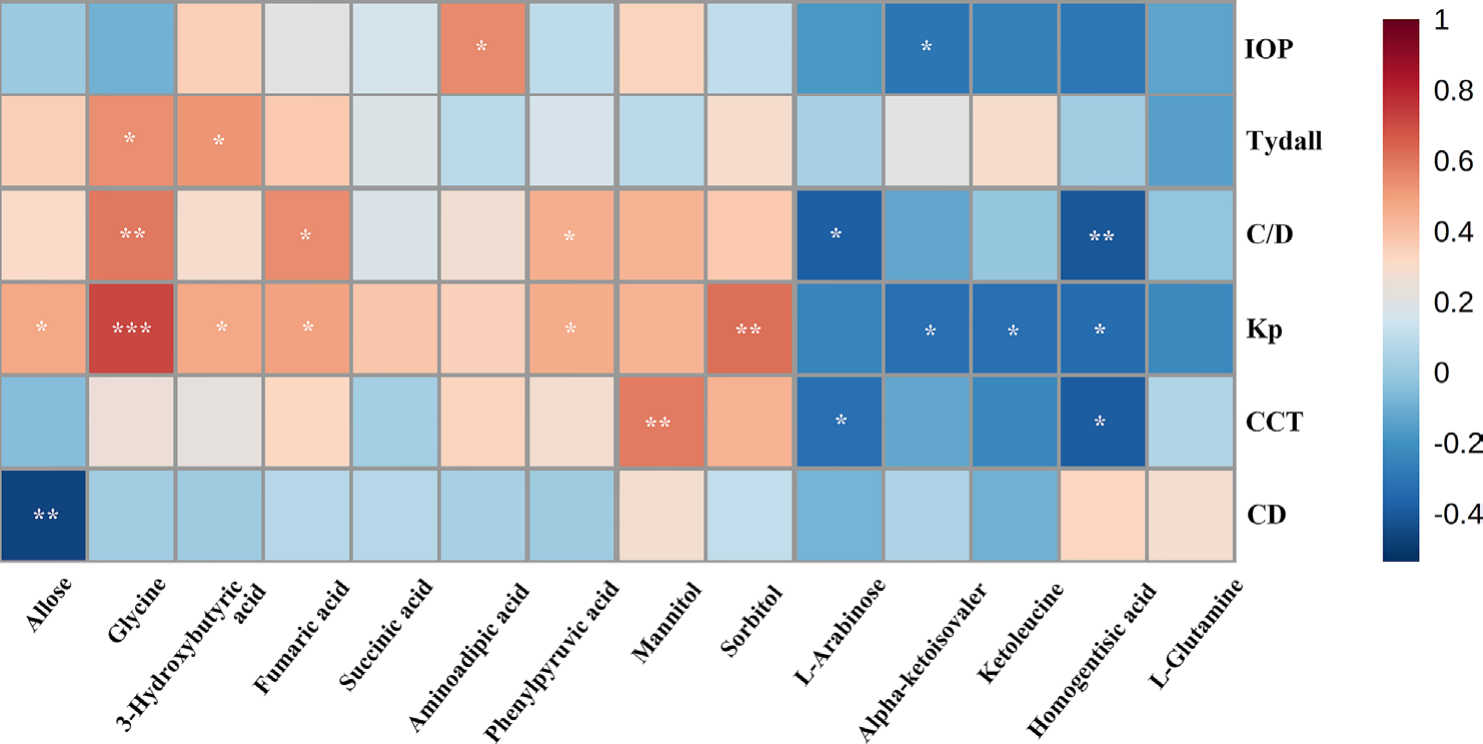
Spearman’s rank correlation analysis. Spearman’s rank correlation analysis performed between 14 different metabolites and clinical indicators. Red, positive correlation; blue, negative correlation. CD, cell density; CCT, central corneal thickness; C/D, ratio of cup to disk; IOP, intraocular pressure; KP, keratic precipitate. *, adjusted *p* value 0.05;**, adjusted *p* value 0.01; ***, adjusted *p* value 0.001.

We evaluated the impact of multiple metabolites and adopted a forward stepwise-regression selection procedure to select the best combination of potential biomarkers for discrimination. The age was adjusted for the logistical regression model. The ROC presentation, on the basis of the logistic regression of metabolic biomarker panel, appears in **Figure 5**. The model containing panel metabolites showed very good discrimination between PSS and control groups. The AUC, sensitivity, and specificity of the logistic regression model established by glycine and homogentisic acid were 1 and 1 (**Figure 5**). Spearman’s rank correlation analysis was conducted among 14 different metabolites with clinical variables. Glycine showed strong positive correlation with Tyndall, C/D ratio, and KP (**Figure 6**). Aminoadipic acid and allose showed positive correlation with clinical indicators, including KP and IOP (**Figure 6**). Alpha-ketoisovaleric acid (KIV), -arabinose, ketoleucine, homogentisic, and allose showed negative correlation with clinical indicators, including IOP, KP, and C/D ratio (**Figure 6**).

## Discussions

Metabolomics is a promising approach for the exploration of disease pathogenesis. The metabolomic profile of AH can directly indicate the physiological status of the eyes (Haines et al., 2018). In our study, 14 metabolites were discovered for the first time from AH samples obtained from patients with PSS—3-hydroxybutyric acid, allose, alpha-ketoisovaleric acid, aminoadipic acid, FA, glycine, homogentisic acid, ketoleucine, -arabinose, -glutamine, mannitol, phenylpyruvic acid, sorbitol, and succinic acid. Some of these metabolites have been recognized as node molecules in pathways associated with inflammation or neuroprotection (Effenberger-Neidnicht et al., 2014; Chen et al., 2016; Xu et al., 2018; Liu et al., 2019). To advance our understanding of the pathophysiological patterns of these identified metabolites, we performed metabolic pathway analysis to identify pathway-based metabolomic features of these metabolites in PSS.

In our research, the lysine degradation metabolic pathway was significantly enriched in PSS (https://www.kegg.jp/kegg-bin/show_pathway?map00310). This enrichment was associated with the concomitant increase of glycine in the samples. Glycine, as a nutritionally important amino acid (AA), is highly essential for fetal and neonatal growth and development (Wang et al., 2014; Wu, 2014). Besides the generation of energy, studies have indicated that glycine can prevent the overproduction of pro-inflammatory cytokines (Li et al., 2001; Stoffels et al., 2011; Effenberger-Neidnicht et al., 2014). Exogenous administration of glycine can modulate the toll-like receptor-4 (TLR4) and nucleotide-binding oligomerization domain protein inflammatory signaling pathway and subsequently downregulate the mRNA expression of related cytokines, such as interleukin-1β (IL-1β), tumor necrosis factor-α (TNF-α), nuclear factor kappa-B (NF-κB) p65, and, especially, the mediator of macrophage recruitment, intercellular adhesion molecule-1 (ICAM-1), which regulates macrophage polarization and recruitment (Wheeler et al., 2000; Stoffels et al., 2011; Xu et al., 2018). We speculated that modulation of the unfavorable local resident macrophages by exogenous administration or endogenous upregulating of glycine might provide a valuable approach to mitigate macrophage-mediated inflammation in PSS. We also know that certain kinds of PSS are caused by HCMV infection. Researches have demonstrated that HCMV infection can induce the activation of the transcription factor NF-κB, which is critical for transactivation of the major immediate-early promoter for HCMV[36]. Hence, downregulation of NF-κB by glycine might be more efficient for treating HCMV-positive PSS. Spearman’s rank correlation analysis confirmed significant strong positive correlation between anti-HCMV IgG and glycine (**Figure 3**). Based on the significant positive correlation between anti-HCMV IgG and glycine, we speculated that glycine might be a bioprobe for HCMV-derived PSS. In this circumstance, combined anti-HCMV therapy might be taken into consideration. Moreover, glycine has been well-documented as having the best neuroprotective effect among all AAs. Accumulated evidence indicates a direct role of glycine in neuroprotection and amelioration of hypoxia-ischemic brain injuries in adult and neonatal animals (Wheeler et al., 2000; Li et al., 2001; DeMeritt et al., 2004; Stoffels et al., 2011; Wang et al., 2014; Wu, 2014; Chen et al., 2016; Mori et al., 2017; Liu et al., 2019). This neuroprotective effect of glycine involved the suppression of inflammatory pathway associated with TNF-α or NF-κB p65/hypoxia inducible factor-1α (Hif-1α) signalling (Wheeler et al., 1999; Liu et al., 2019). Stroke patients clinically treated with glycine-based modalities demonstrated favorable outcome and a reduced tendency for 30-day mortality (Gusev et al., 2000). Similar to cerebrospinal fluid, increased amount of glycine was detected in the AH of patients with PSS, which might confer a neuroprotection from optic neuropathy caused by marked elevation of IOP during the acute phase of PSS.

KIV was found decreased in the AH of patients with PSS. The associated valine–leucine–isoleucine biosynthesis pathway was highlighted during the *in silico* pathway analysis as an important pathway in PSS (https://www.kegg.jp/kegg-bin/show_pathway?map00290). Valine, leucine, and isoleucine from this pathway constitute the branched-chain AAs (BCAAs) family. BCAAs are strong nutritional stimuli that are able to regulate the inflammatory status by the regulation of glutamine production (Nicastro et al., 2012; Gameiro and Struhl, 2018). Under inflammatory circumstances, BCAAs can be transaminated to glutamate to meet the high requirement of glutamate in inflammatory cells, such as macrophages, and help maintain the cellular function (Pithon-Curi et al., 2004). Moreover, BCAAs have been widely known to activate mammalian target of rapamycin (mTOR), which can modulate autophagy and inflammatory responses (Xu and Brink, 2016; Saxton and Sabatini, 2017; Sinclair et al., 2017). Reports have demonstrated that mTOR can facilitate PI3K/Akt-induced NF-κB activation (Peppenelli et al., 2018; Altman et al., 2019), an important step in all the three stages of HCMV infection (Li et al., 2015; Peppenelli et al., 2018; Altman et al., 2019) (https://www.kegg.jp/kegg-bin/highlight_pathway?scale=1.0&map=map05163&keyword=human%20herpesvirus%203). Consequently, targeting the PIK/Akt/mTOR cascade might be more efficient for treating HCMV-positive PSS. Significantly decreased KIV was found in the AH of PSS. We speculated that KIV uptake increased significantly during the course of HCMV infection. Mammalian mitochondrial branched-chain α-keto acid dehydrogenase complex can catalyze the oxidative decarboxylation of KIV to yield the BCAA product, valine (Funchal et al., 2007). The metabolic stimulation of this step may be responsible for the decrease in KIV and accumulation of BCAA. This information provides a target for the metabolic inhibition of this step, which might reverse the result and diminish the activity of BCAAs and PIK/Akt/mTOR cascade.

Besides the PIK/Akt/mTOR pathway, BCAAs-induced mTOR activation can also significantly contribute to the upregulation of vascular endothelial growth factor (VEGF) level (Brugarolas et al., 2003; Klos et al., 2006), which has been found to be highly expressed in the AH of patients with uveitic glaucoma (Ohira et al., 2016). Recently, emerging evidences indicate that the VEGF–VEGF receptor (R) system is significantly involved in inflammation (Shibuya, 2015). Among the VEGFRs, VEGFR1 is expressed on the membrane of macrophage lineage cells and mediates the transduction of important cytokine/chemokine signaling in these cells (Shibuya, 2015). This VEGFR1–macrophage axis can trigger inflammatory responses in various tissues and might be a potential target for managing inflammation. Taken together, we surmised that the inhibition of BCAA/mTOR pathway could have beneficial therapeutic effects by preventing inflammation-mediated pathogenesis.

Marked increase in succinic acid and the associated pathways, citrate acid cycle (tricarboxylic acid [TCA] cycle) (https://www.kegg.jp/kegg-bin/show_pathway?map00020) was detected in the PSS group. TCA cycle has been considered to interact with the frontline innate leukocytes, such as macrophages and monocytes (Jha et al., 2015; Kelly and O'Neill, 2015; Murphy and O'Neill, 2018). The mechanism behind this process might involve the metabolic reprogramming of endogenous TCA cycle intermediates, namely, citric acid, which adopts regulatory features to harness inflammatory response. Typically, in the TCA cycle, citrate is converted to isocitrate, which is then diverted to succinate, a key marker of macrophage activation. Succinate has been found to be elevated in metabolic disorders associated with inflammation (Tannahill et al., 2013; Mills and O'Neill, 2014). Intracellular succinate accumulation is postulated to regulate the IL-1β–HIF-1α axis to exert pro-inflammatory function (Tannahill et al., 2013; Mills and O'Neill, 2014; Lampropoulou et al., 2016; Xiao et al., 2017). Meanwhile, we also detected increased FA in PSS AH, another well-known intermediate product of TCA. Recently, FA has shown its therapeutic potentials for the management of inflammatory clinical diseases, such as autoimmune myocarditis and multiple sclerosis (Wakkee and Thio, 2007; Meili-Butz et al., 2008; Moharregh-Khiabani et al., 2009; Loewe et al., 2011). Hence, the regulation of TCA to generate favorable metabolites may have important clinical utility for the treatment of PSS.

Besides pathway analysis, ROC analysis was conducted to evaluate the diagnostic potential of selected metabolites. The results of ROC analysis implied that the combination of glycine and homogentisic acid could serve as potential biomarkers for the discrimination of control and PSS groups. Spearman’s rank correlation analysis simultaneously revealed that these two metabolites, differentially expressed in patients with PSS, presented significant correlation with IOP, C/D, and anti-HCMV IgG titer in AH (**Figures 3** and **6**). This discovery demonstrated immense potential for PSS diagnosis. Most importantly, these metabolites could also provide valuable information regarding subtype identification and allow us to perform specific intervention for patients with PSS.

In conclusion, these results revealed for the first time the identity of important metabolites and pathways contributing to the development/progression of PSS. These discoveries enabled the better understanding of the mechanism of PSS and might lead to the development of metabolic biomarkers and novel therapeutic strategies to restrict the development/progression of PSS. Further investigations are needed to validate the role of these metabolites and relevant metabolic reprogramming pathways. We have realized there are limitations in our research. The following studies will recruit more participants and choose more suitable control group to support our results.

## Data Availability Statement

The raw data supporting the conclusions of this manuscript will be made available by the authors, without undue reservation, to any qualified researcher.

## Ethics Statement

The studies involving human participants were reviewed and approved by the medical ethics committee of the Eye, Ear, Nose and Throat (ENT) Hospital of Fudan University (2017006–2). The patients/participants provided their written informed consent to participate in this study.

## Author Contributions

HW designed the study, evaluated of the results, and wrote the draft and final manuscript. RZ collected the clinical data of the subjects and analyzed statistical data. QS clinically evaluated the subjects and collected the AH sample. YW collected the metabolomic data. ZW performed the laboratory exams. JF evaluated of the original metabolomic data and edited the draft manuscript. XK evaluated the clinical characteristics of the subjects to establish the diagnosis, performed clinical procedures of the study, and edited the final manuscript. All authors approved the final manuscript.

## Funding

This work was supported in part by grants from the National Natural Science Foundation of China (81870666), National Key R&D Program of China (2016YFC0904800), the Surface Project of National Natural Science Foundation of China (81770922), the project of Shanghai Municipal Commission of Health and Family Planning (201740204), the clinical science and technology innovation project of Shanghai Shenkang Hospital Development Center (SHDC12017X18), and the western medicine guidance project of Shanghai Committee of Science and Technology (19411961600). The open access publication fees were supported by the National Natural Science Foundation of China (81870666). The funders had no role in study design, data collection and analysis, decision to publish, or preparation of the manuscript. The authors declared that no conflicts of interest exist.

## Conflict of Interest

The authors declare that the research was conducted in the absence of any commercial or financial relationships that could be construed as a potential conflict of interest.

## Supplementary Material

Figure S1 The validation plot from the 200-permutation test model. The Q2Y of the original model on the right were higher than the corresponding values of the permutaion test models on the left.
