# Supplementary figures and images for: Metabolomic Profile of Posner–Schlossman Syndrome: A Gas Chromatography Time-of-Flight Mass Spectrometry-Based Approach Using Aqueous Humor
Source: Front Pharmacol. 2019 Nov 7;10:1322. doi: 10.3389/fphar.2019.01322 (PMC6855217; doi:10.3389/fphar.2019.01322)

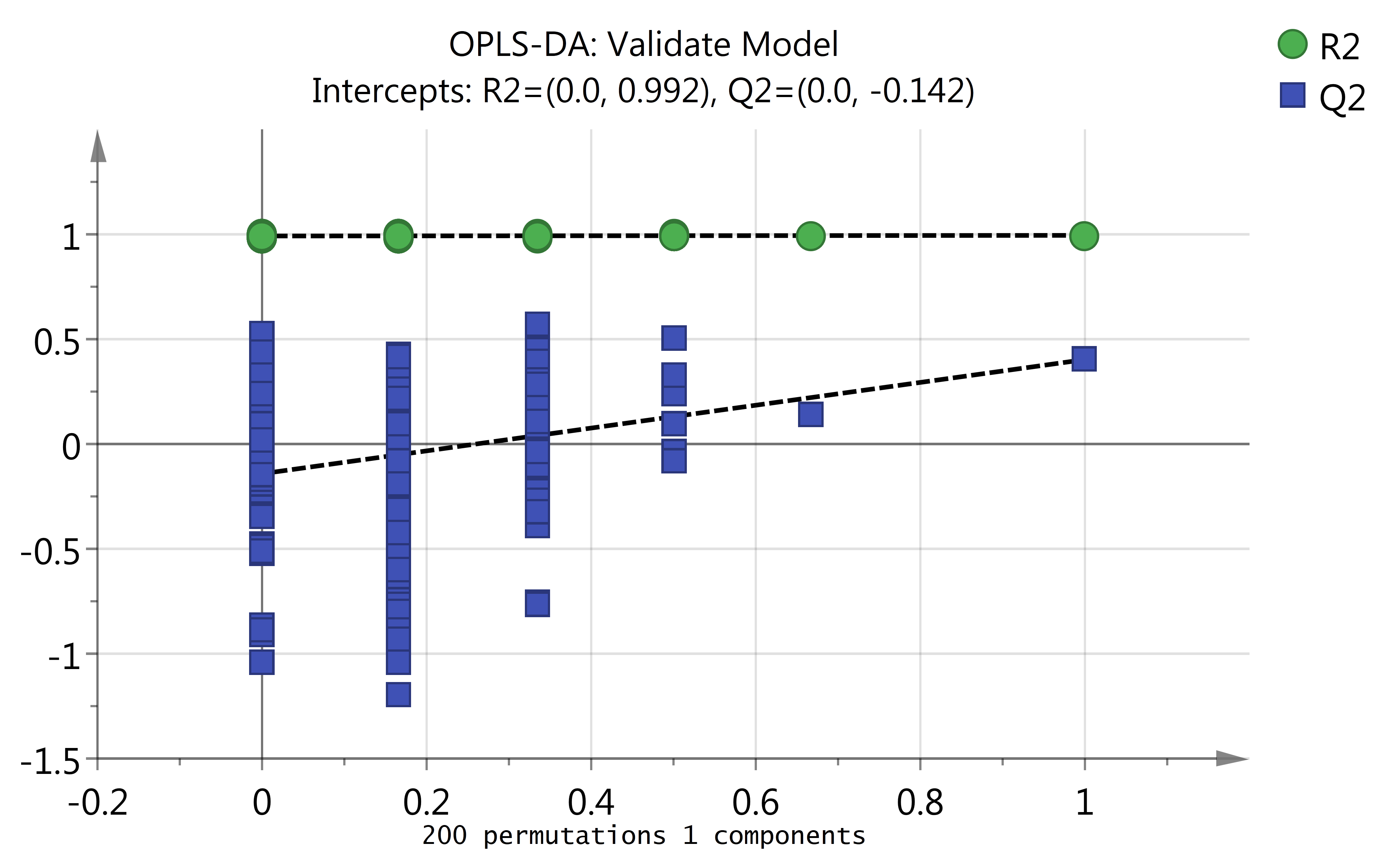

Supplement: Figure S1 — The validation plot from the 200-permutation test model. The Q2Y of the original model on the right were higher than the corresponding values of the permutaion test models on the left. [file Image_1.tif]
