# Supplementary material for: Metabolomic Profile of Posner–Schlossman Syndrome: A Gas Chromatography Time-of-Flight Mass Spectrometry-Based Approach Using Aqueous Humor
Source: Front Pharmacol. 2019 Nov 7;10:1322. doi: 10.3389/fphar.2019.01322 (PMC6855217; doi:10.3389/fphar.2019.01322)
Supplement: Supplementary file 2 [file Table_1.docx]

***Supplementary Methods:***

***Sample preparation procedure***

The samples were thawed on ice-bath and centrifuged for 5 min at 4 ºC and 3,000 g (Microfuge 20R, Beckman Coulter, Inc., Indianapolis, IN, USA) to separate debris or a lipid layer. Each sample aliquot of 50 μL was mixed with 10 μL of internal standard, to which 175 μL of pre-chilled methanol/chloroform (v/v=3/1) were added. After the mixture was kept at -20 ºC freezer for 20 min and centrifuged at 14,000 g and 4 ºC for 20 min, the supernatant was carefully transferred to an autosampler vial (Agilent Technologies, Foster City, CA, USA). All the samples in autosampler vials were evaporated briefly to remove chloroform using a CentriVap vacuum concentrator (Labconco, Kanasa City, MO, USA), and further lyophilized with a FreeZone freeze dryer equipped with a stopping tray dryer (Labconco, Kansas City, MO, USA).

The sample derivatization and injection were performed by a robotic multipurpose sample MPS2 with dual heads (Gerstel, Muehlheim, Germany). Briefly, the dried sample was derivatized with 50 μL of methoxyamine (20 mg/mL in pyridine) at 30 ºC for 2 hr, followed by the addition of 50 μL of MSTFA (1% TMCS) containing FAMEs as retention indices and further incubation at 37.5 ºC for another 1 hr using the sample preparation head. In parallel, the derivatized samples were injected with sample injection head after derivatization.

***Mass-spectrometry analysis***

A time-of-flight mass spectrometry (GC-TOF/MS) system (Pegasus HT, Leco Corp., St. Joseph, MO, USA) with an Agilent 7890 B gas chromatography and a Gerstel multipurpose sample MPS 2 with dual heads (Gerstel, Muchlheim ,Germany). A Rxi-5 ms capillary column (30m×250 μm i.d., 0.25-μm film thickness; Restek corporation, Bellefonte, PA, USA) was used for separation. Helium was used as the carrier gas at a constant flow rate of 1.0 mL/min. Derivatized samples of 1μL each were injected into GC/MS instrument in splitless injection mode. A programmed column temperature was optimized for successful separation (Table S1). The temperature of injection and transfer interface were both set to 270 ºC. The source temperature was 220 ºC. The measurements were made using electron impact ionization (70 eV) in the full scan mode (*m/z* 50-500). Acquisition rate was set to 25 spectra/sec. Instrument optimization was performed every 24 hours.

Table S1. Temperature program of column incubator in GC-TOFMS

| Rate (ºC min^-1^) | Temperature (ºC） | Hold time (min) |
| --- | --- | --- |
|  | 80 | 2 |
| 12 | 300 | 0 |
| 0 | 300 | 4.5 |
| 40 | 320 | 0 |
| 0 | 320 | 1 |
